# Dosimetry Comparison of Palliative Radiation Plans Generated From Available Diagnostic CT Images Versus Dedicated CT Simulation for Inpatients

**DOI:** 10.7759/cureus.17799

**Published:** 2021-09-07

**Authors:** Quoc-Anh Ho, Lexie Smith-Raymond, Angela Locke, Jared R Robbins

**Affiliations:** 1 Radiation Oncology, The University of Arizona College of Medicine – Tucson, Tucson, USA; 2 Radiation Oncology, Banner - University Medical Center Tucson, Tucson, USA

**Keywords:** radiation oncology, palliative, radiation, medical physics, dosimetry, spinal cord compression

## Abstract

Introduction

The morbidity sequelae of advanced cancer are often irreversible. Early palliative radiation can prevent, delay, and even improve these consequences. Treatment may be delayed due to a packed computed tomography (CT) simulation schedule or other logistics, including the cost and burden of arranging ambulance transportation when radiation centers are off-site.

Objectives

The primary objective was to determine the feasibility of using a recent diagnostic CT scan in lieu of a dedicated simulation CT to generate an adequate plan without sacrificing dosimetric goals and subsequent efficacy or tolerability. Secondary objectives included how much the lesion has grown, and how much earlier treatment could start if planned on a diagnostic CT scan.

Materials/Methods

For each inpatient treated with palliative radiation, a prior recent diagnostic CT scan was imported into the RayStation (RaySearch Laboratories, Stockholm, Sweden) planning system. From these diagnostic scans, planning treatment volumes (PTV) and organs at risk (OAR) were contoured using the same technique as the patient’s actual treatment. The primary outcome was to compare both the PTV coverage and OAR dose between the plan generated from the diagnostic CT compared to that from the simulation CT. Our secondary outcomes include the mean time between CT simulation and first treatment, change in tumor volume between diagnostic scan and CT simulation, and the hottest 1% of each plan (D1).

Results

Between May and August 2019, a total of 22 inpatients were treated palliatively. Of those 22 patients, 10 patients (ages 32-92 years, median 64.5 years, 50% spine) met study criteria and had a diagnostic CT scan that was obtained within 14 days of simulation CT that was also compatible with our planning software. In the plans that were delivered, a mean of 98.8% (range 94.4-100%) of PTV was covered by at least 95% prescription dose. In the diagnostic CT plans, a mean of 95.4% (range 84.5-100%) of PTV was covered by at least 95% prescription dose. The difference between plans trended towards significance (p=0.061). When looking at patients receiving treatment to the spine or having a diagnostic CT within four days of the simulation CT, there was no statistically significant difference between the two plans (p=0.032 and 0.030, respectively). The OARs received, on average, 1.4% less mean radiation dose in the hypothetical plans (p=0.911). All OAR constraints were met in both groups. The mean time between diagnostic CT and CT simulation was 5.9 days and between CT simulation and first treatment was 1.9 days (range 0-5 days). The mean change in tumor volume was 22.64% smaller in the diagnostic CT scan plan. The D1 was an average 1% hotter in the hypothetical plans (p=0.16).

Conclusion

In hospitalized patients with an indication for palliative radiation, treatment planning on a pre-existing recent diagnostic CT scan produces comparable dose distributions without increases in dose to OARs when compared to the use of CT simulation scans, particularly for the treatment of the spine or when a very recent diagnostic CT is available. Bypassing CT simulation in select cases allows for earlier delivery of radiation with less patient and logistical burden. In combination with daily image guidance, this may translate to more timely delivery of radiation, less cost and burden to critically ill patients, and improved palliative benefit.

## Introduction

The sequelae of advanced stages of cancer are serious and often irreversible. These sequelae include but are not limited to malignant spinal cord compression (mSCC), cauda equina syndrome, airway obstruction, superior vena cava (SVC) syndrome, and significant pain. These severe consequences are a major cause of morbidity and mortality in this patient population. Early palliation can prevent, delay, or even improve these consequences. Radiation therapy is an established palliative treatment for these conditions and is often delivered urgently in the inpatient setting [1].

Radiation therapy has evolved significantly in the past few decades, which has allowed practitioners to escalate doses to improve local control in the definitive setting. One major advent of the field is the use of computed tomography (CT) scans to deliver radiation as 3D conformal radiation therapy (3D-CRT). This process begins with a simulation CT to allow for treatment planning. The purpose of CT simulation is to improve the accuracy of treatment and lower toxicity [2]. This has allowed higher doses of radiation to be used, which subsequently increases local control.

The benefits of CT simulation and dose-escalation using 3D-CRT is evident in the curative setting and have carried over to the palliative setting, even though palliative radiation requires much lower doses of radiation than definitive radiation. Despite the adoption of CT simulation and 3D-CRT in the palliative setting, the standard doses of palliative radiation have remained largely unchanged, and in some cases, have decreased [3]. As such, CT simulation may not be necessary in many palliative cases. Furthermore, CT simulation does require additional logistical consideration. Appropriate personnel and open time slots for the CT simulator may not be readily available. Furthermore, many radiation centers are not hospital-based, so for hospitalized patients, initial, dedicated ambulance transportation for CT simulation is necessary.

The primary purpose of this study was to determine if by using a recent diagnostic CT scan in lieu of a dedicated simulation CT, a comparable plan could be produced without sacrificing dosimetric goals and subsequent efficacy or tolerability. Secondary objectives investigated how much the lesion has grown, and how much earlier treatment could start if planned on a diagnostic CT scan. We hypothesize that planning on a diagnostic CT scan would produce an equally safe and effective plan while expediting the initiation of radiation and sparing one transportation to the radiation oncology department.

This study was accepted for oral presentation at the American Radium Society Annual Meeting 2020 and to be presented at the American Radium Society Annual Meeting 2021.

## Materials and methods

Patients and treatment

In this single-institution retrospective review, we analyzed all patients treated palliatively while hospitalized between May and August 2019. Approval by the institutional review board was obtained (The University of Arizona IRB, protocol number 2107068548). Inclusion criteria were as follows: CT simulation occurred during hospitalization, the first fraction of radiation occurred during hospitalization, diagnostic CT that is no older than 14 days was available at the time of CT simulation, and the diagnostic CT was compatible with planning software.

Treatment planning

Patients who met the criteria had their most recent diagnostic CT scan imported into the RayStation (RaySearch Laboratories, Stockholm, Sweden) planning system. A resident and attending radiation oncologist contoured the gross tumor volume (GTV), planning tumor volume (PTV), and organs at risk (OAR) based on the palliative goals and margins used during the actual treatment. These diagnostic CT scan contours were then planned by two dosimetrists who used the same 3D-CRT planning approach as was done on the simulation CT scan. The plan generated on the diagnostic CT scan was then transferred directly onto the simulation CT for evaluation of the coverage and constraints. As the simulation CT was the most recent imaging prior to the first treatment, transferring the diagnostic plan onto the simulation CT served as a surrogate for how accurate the hypothetical plan would be.

Statistical analysis

**Figure 1:**
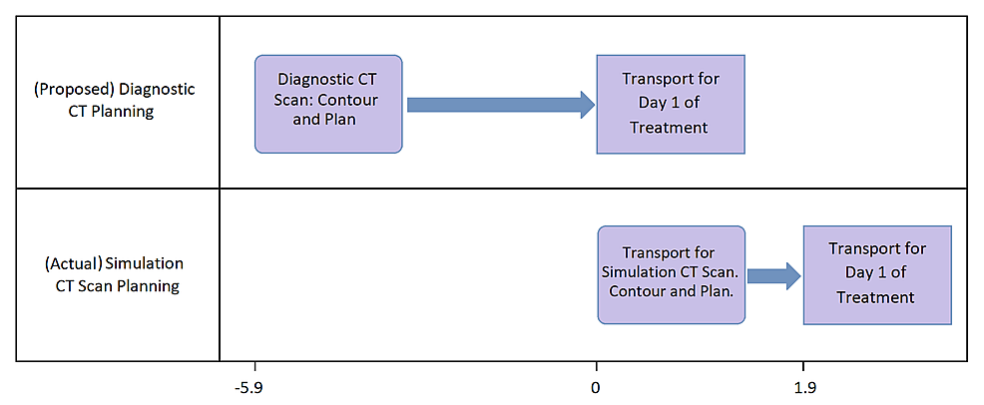
The proposed planning on the diagnostic CT scan potentially expedites the start of treatment by 1.9 days, on average.

The primary outcome was to compare the PTV coverage and OAR dose between the plan generated from contouring the diagnostic CT but evaluated on the simulation CT and that from the plan directly contoured and delivered onto the simulation CT. The contours and planning from the diagnostic CT were done entirely for dosimetric comparison and analysis; no radiation was delivered based on the newly generated plan. The specific metric for PTV coverage used was the mean % PTV volume that received 95% of prescription dose (D95%) of each group. Pre-selected OARs were used depending on the vicinity and likely clinical significance of the received dose. The mean radiation to each OAR was obtained and compared from the diagnostic CT to the simulation CT scan. Secondary outcomes include the mean time between CT simulation and first treatment, change in tumor volume between diagnostic scan and CT simulation, and the hottest 1% of each plan. Change in tumor volume was only obtained for non-spine disease sites, as it was more easily quantifiable. As the date of CT simulation is often the earliest transportation can be arranged, this date also served as a surrogate for the first treatment date in the comparison hypothetical diagnostic CT plan. We used the one-sided t-test to determine if there was a statistically significant decrease in PTV volume that received 95% of prescription dose when planning on the diagnostic CT compared to the simulation CT. The null hypothesis that each metric did not have a statistically significant decrease when using the diagnostic CT was rejected if p<0.05. Figure 1 represents how data and planning for the two groups were collected.

## Results

Patients

**Figure 2:**
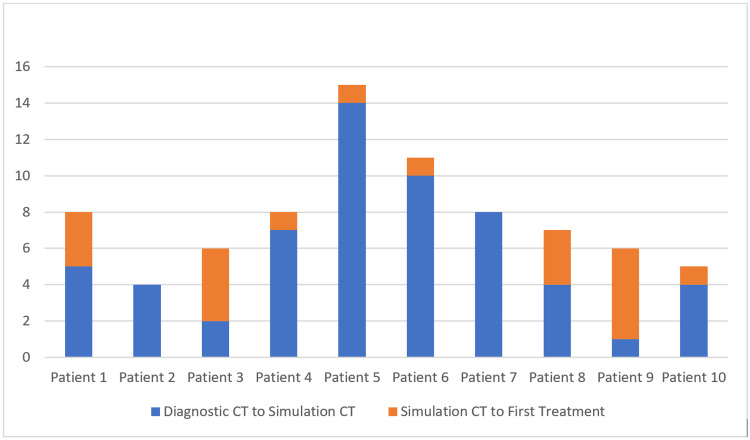
Stacked bar graph representing the time (in days) between the most recent diagnostic CT to CT simulation and from CT simulation to the first fraction

Of the 22 patients reviewed, 10 (45.5%) met eligibility criteria. Of those excluded, one had their CT simulation and subsequent planning done prior to hospitalization, five did not have a diagnostic CT within the 14-day window prior to CT simulation, and six had a diagnostic CT that was not in a format compatible with import into RayStation. The reasons for RayStation incompatibility were: differing pitch angles, resizing and saving images outside of root digital imaging and communications in medicine (DICOM) format, and/or unequal image slice thickness. The remaining 10 patients (ages 32-92, median = 64.5) had their diagnostic CT scan imported into RayStation. Of these 10 patients, 50% had spinal cord or cauda equina compression, 30% had respiratory compromise from a lung mass, and 20% had a non-spine bone (chest wall or iliac) mass. All patients reviewed received their prescribed radiation and had no greater than grade 2 acute toxicity. The time from most recent diagnostic CT to simulation CT ranged from 1 to 14 days with a mean of 5.9 days. The time from simulation CT to the first fraction ranged from zero to five days with a mean of 1.9 days. Mean hospitalization duration of 27.2 days (range = 10-88 days), although this mean dropped to 20.4 days (range = 10-37 days) if we omitted a single patient who presented for an unrelated illness, with consultation occurring on day 80 and death on day 88. Table 1 summarizes the patient characteristics of those eligible. Figure 2 represents the timing between each step for individual patients.

**Table 1:** Patient characteristics

Age	Primary Cancer	Site of Treatment	Treatment Regimen	Length of Hospitalization (days)	Time from Diagnostic CT to Simulation CT (days)	Time Simulation CT to First Fraction (days)
73	Squamous Cell Carcinoma of Lung	Right Lung	20Gy/5fx	10	5	3
59	Cutaneous Melanoma	Lumbar Spine	8Gy/1fx	31	4	0
68	Squamous Cell Carcinoma of Lung	Right Lung	20Gy/5fx	17	2	4
53	Leiomyosarcoma	Left Lung	14.4Gy/4fx BID	14	7	1
76	Multiple Myeloma	T11	8Gy/1fx	21	14	1
62	Urothelial Carcinoma	Left Chest Wall	8Gy/1fx	19	10	1
32	Leiomyosarcoma	Lumbosacral Spine	25Gy/5fx	24	8	0
67	Urothelial Carcinoma	Iliac Bone	8Gy/1fx	88	4	3
60	Squamous Cell Carcinoma of Esophagus	Lumbar Spine	20Gy/5fx	37	1	5
92	Prostate	T10-L3	8Gy/1fx	11	4	1

Within the group of plans created from a simulation CT, a mean of 98.8% (range = 84.41-100%) of PTV received at least 95% of the prescription dose. The group of plans created from a diagnostic CT had a mean 96.1% (range = 84.5-100%) of PTV covered by at least 95% of the prescription dose. There was not a statistically significant decrease in PTV coverage, although there was a trend towards significance (p=0.061). Of note, the three individual subjects who had the most decrease in PTV coverage had between 10% and 15.5% decrease in coverage. These three also had non-spine targets (2 lung, 1 chest wall) and their time from diagnostic to simulation CT was five, two, and 10 days, respectively. When looking only at spine lesions, the means were 99.41% (range = 97.45-100%) for the diagnostic CT planned group, versus 98.81% (range = 94.41-100%) for the simulation CT planned group. There was no significant decrease in coverage from using these diagnostic CT scans (p = 0.32). When looking at the five patients who had a diagnostic CT scan within four days prior to the simulation CT, the means were 97.15% (range = 89%-100%) for the diagnostic CT scan, versus 98.38% (range = 94.41%-99.97%) for the simulation CT scan. There was no significant decrease in coverage from using these diagnostic CT scans (p = 0.30).

Secondary outcomes

**Figure 3:**
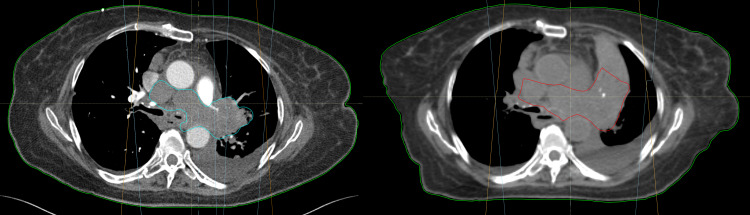
Diagnostic CT with GTV volume (left) compared to simulation CT with GTV volume (right) In this particular patient, there was a 21.8% increase in volume between the two scans. GTV: gross tumor volume

**Figure 4:**
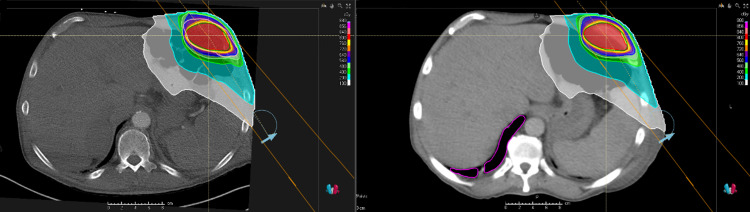
Plan evaluation of diagnostic CT and simulation CT Although the plan created on diagnostic CT that was transposed to simulation CT only had 86% PTV coverage, we suspect this is due to there being a 10-day delay between diagnostic CT and simulation CT. As seen on the left image of planning and evaluation both on diagnostic CT, coverage is excellent when not transferred to CT simulation. PTV contours for the diagnostic scan (peach) and simulation scan (green).

**Figure 5:**
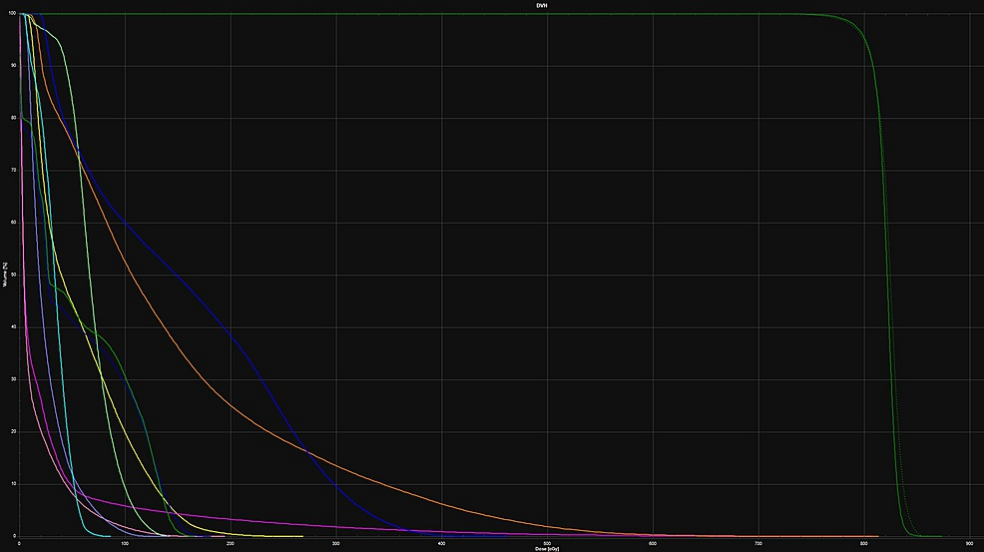
Dose-volume histogram for the patient in Figure 4, treated with 8Gy/1fx The X-axis represents dose and Y-axis represents % volume of target or OAR. For simulation CT: PTV (solid green on far right), left kidney (blue), spinal cord (dark blue), stomach (light green), liver (lavender), heart (light pink). For diagnostic CT: PTV (dotted green), left kidney (orange), spinal cord (solid green on left), stomach (yellow), liver (light blue), heart (magenta). OAR: organs at risk; PTV: planning treatment volume

The OARs received an average of 1.4% less absolute radiation at the prescription dose in the diagnostic CT plan but this was not statistically significant (p=0.911). All OAR constraints were met in both groups. The mean change in GTV was a 22.64% (median 17.6%, range 0.4-60%) increase in the simulation CT scan plan. This represents an average growth of about 3.5% growth per day (median 1.8% growth per day, range 0.2-10.4% per day). Of note, the two fastest-growing lesions were both in the lung and grew about 10% per day. Figure 3 shows a patient who had had a lung lesion that grew 21.8% between diagnostic and simulation CT. Overall, the D1 was an average 1% hotter in the diagnostic CT plans but this was not statistically significant (p=0.16). Figure 4 shows a patient who would have had excellent PTV coverage if planning and evaluation were both done on a diagnostic CT scan. There was a median survival of 66.5 days (range 3 to 374 days) from the time of the first actual fraction of radiation. Figure 5 shows the dose-volume histogram for the patient in Figure 4.

## Discussion

Our study shows equivalent PTV coverage between plans generated from a recent diagnostic CT compared to a dedicated CT-simulation scan, although there was a trend towards a decrease in PTV coverage when planning from a diagnostic CT scan. These slight differences are likely the result of slight differences in anatomy and patient positioning between the diagnostic CT and the simulation CT that accounts for the trend to lower coverage. In the hypothetical plan, the contouring and planning were done on the diagnostic CT and evaluated on the simulation CT. This was done as the simulation CT would have had the anatomy that most closely resembled day one of treatment, whereas the actual plan delivered was contoured, planned, and evaluated all on the same simulation CT scan, for which the anatomy never changed. As our goal was to see if planning on a diagnostic CT was feasible and comparable, we felt that our approach was appropriate. Similar studies have been performed that came to the same conclusion. Glober et al. evaluated 25 patients (10 heterotopic ossification, 10 spinal metastases, and 5 lung lesions) with diagnostic scan-based planning and found a median D95% coverage of 96% (range 86-100%) with a median Dmax of 108% (range 102-113%). Of note, this study also commented on lung plans having a lower D95 of 94% and an unspecified higher hot-spot. There was no indication of when the diagnostic CT was taken relative to the simulation CT date. They subsequently treated 10 patients prospectively and found that the median time from check-in to completion of radiation was 28 minutes [4]. A separate study by Wong et al. reviewed 150 diagnostic CT scans along with their respective simulation CT scans, and found that treatment to the pelvis, abdomen, and thoracic and lumbar spines were the most reproducible. They created a full-body vac-bag to replicate the curvature of a diagnostic CT and treated 30 patients prospectively. They found that Hounsfield unit variations between the two scans were greatest in the thorax, and that the 95% dose coverage (V95) varied between -2% and 2.5% [5]. Finally, a third, separate trial by Schuler et al treated 150 patients with pain from osseous or soft-tissue metastases, without CT-simulation and noted that the rates of pain response, including pain resolution, were not significantly different from randomized historical controls [6]. Our findings support the previous data that diagnostic CT-based planning is safe and efficacious dosimetrically and that treatment of the lung is less ideal than other sites. However, our analysis looked deeper into the optimal timing of usage of a diagnostic CT as well as the potential expedition of treatment and shortening of hospital stay by bypassing CT simulation. When looking specifically at patients who had their diagnostic CT within four days before their simulation CT, or those with a non-lung site, the trend towards a significant difference disappeared. As spine treatments target the entire vertebral body(s) as the clinical target volume (CTV), it is highly unlikely that the number of involved vertebral bodies, and, subsequently, the CTV and GTV, would change within a few days. Similarly, patients with a more recent diagnostic CT scan would have had less time to have their tumor grow, and there would subsequently be less of a difference in anatomy. Furthermore, lung lesions in this study grew the fastest, and this could explain why their omission led to more equal coverage. Radiation is well-established as an effective treatment for many solid malignancy emergencies. However, patients with cancer are well-known to have prolonged hospital stays and increased cost of hospitalization of 32% longer and 53% costlier, respectively [7]. Much of the reason for this increased cost and duration is due to the complexity of cancer patients, as many have a poor functional performance status and subsequently are more difficult to discharge safely. However, radiation is also a major factor behind these increased values. In a study by Pintova et al., a single-institution retrospective review, hospitalized cancer patients receiving radiation were found to have a hospitalization that is seven times, or about 33 days, longer than hospitalized cancer patients who did not receive radiation. When this value was evaluated for the duration attributed to radiation treatment, it was still 11 days longer than patients not receiving radiation. These patients who received radiation also exceeded their expected length of stay by over 400%. In that study, the charge of radiation therapy was calculated to be $12,514 per patient. However, the increase in the total charge of each patient was $44,676 higher than the expected charge when factoring in all costs of prolonged hospitalization. The study does not specifically address non-radiation factors that prolonged hospitalization but does emphasize that some patients preferred finishing radiation while hospitalized, as they did not feel safe returning home, even when assessed to be safe by physical therapy. The study also addressed transportation issues for outpatient radiation and underutilization of single fraction treatment. It also found that disposition to skilled nursing facilities was difficult with active radiation treatment, which factored into the longer hospitalization [8]. Similar findings were also found by Cushman et al., who showed patients treated with radiation within two days of admission had a mean of six days of hospitalization versus a mean of 13 days for those who did not start their radiation within two days of admission [9]. The same findings can be extrapolated from our study, which showed that hospitalized patients receiving radiation ended up staying on average 27 days (20 days if we removed outlier), which is longer than patients not receiving inpatient radiation in the above-cited studies. At our institution, one ambulance trip costs $2500, and one day of hospitalization, at minimum, also costs $2500. The ‘back-end’ factors that prolong hospitalization and delay discharge in our cohort is beyond the scope of this study. Our focus was primarily on the ‘front end’ of the hospitalization, specifically how CT simulation contributes to the delay in initiating radiation and increases costs. The results of this study show that target volume dose and OAR constraints were not sacrificed when planning on a diagnostic CT as opposed to a dedicated simulation CT. Differences between plans generated from a diagnostic CT and the simulation CT were negligible when selecting optimal patients like those with spine metastases and recent diagnostic CTs. Bypassing a simulation CT in these select cases would allow one less trip to the radiation facility, which is safer, and saves patient discomfort, time, and other resources. This becomes especially beneficial when the radiation facility is in a remote location, which requires ambulance transportation to reach. Furthermore, limiting trips also reduces the spread of infectious diseases or the probability of the patient becoming unstable while en route. As the mean time between simulation CT and first fraction was 1.9 days, and CT simulation is usually scheduled as soon as allowable, these 1.9 days serves as the amount of time potentially saved if planning was performed on the diagnostic CT scan and treatment began on the would have been CT simulation date. Based on our secondary outcomes, there are also clinical benefits to treating on a diagnostic CT scan. As it could take hours or days to coordinate transportation for simulation and first treatment, this allows for extra time for the tumor to grow. Additionally, a larger target would lead to more scatter radiation to OARs. One limitation of our study is the use of diagnostic CT scans of up to 14 days. As the study showed, tumors can grow at a steady pace, and by 14 days, they may no longer resemble how they appeared on the diagnostic CT scan. This timeframe cutoff was used to increase the sample size for the study, but we acknowledge that it would not be stringent enough in an actual treatment setting, as found when there was a trend towards significance in decreased coverage when all CT scans older than four days were used. However, if a CT simulation needs to be bypassed in a real situation, it would not be difficult to coordinate with the inpatient team to have a diagnostic CT performed in the few days before transportation for the first fraction. Furthermore, our study identified some of the common causes of diagnostic scan incompatibility for planning (differing pitch angles, resizing and saving images outside of DICOM format, and/or unequal image slice thickness), which could allow providers to work with hospital radiology to ensure that the diagnostic scan would be compatible. Another limitation is that there are known physical differences between a diagnostic CT bed and a simulation CT bed. Diagnostic CT scanner beds generally have rounded edges, whereas simulation CT scanners beds are completely flat, similar to a linear accelerator couch. If this causes the tumor to not align within the treatment field due to it assuming a slightly different positioning, rotational angle, or shape, then it could reduce the efficacy of palliation if parts of the tumor are undercovered. Furthermore, there could be an increase in toxicity if a larger treatment field is needed to cover the uncertainty of tumor location. This is evidenced by the trend towards a significant decrease in coverage when all 10 patients were evaluated together, but the lack of any decrease when only spine patients were evaluated. These potential shifts can be monitored and addressed through daily image guidance, particularly a cone-beam CT scan, for at least the first fraction. Alternatively, one of the previously mentioned studies overcame this difference by using a vacuum-locked body bag molded in a curved shape similar to a diagnostic CT bed [5].

Along with treatment expense and interdepartmental educational deficiencies, the logistics and coordination of inpatient radiation are major barriers to timely treatment [10-11]. Current palliative radiation doses, such as the ones used on the patients reviewed, have been standard-of-care for decades, even before 3D-CRT and CT simulation became widespread. The findings of this study suggest that when a spine metastasis is targeted or a diagnostic CT up to four days old is utilized, the dosimetric outcomes from a plan created from a diagnostic CT scan are not significantly decreased compared to the plan generated from a CT simulation. We conclude that treatment planning on a diagnostic CT is very likely to be safely performed in many inpatient palliative cases. This, combined with palliative and multidisciplinary care, can significantly shorten the length of hospitalization, reduce costs, and potentially improve outcomes. A prospective interventional trial is underway to validate the findings of this study.

## Conclusions

Metastatic cancer is a source of significant morbidity, often requiring long, expensive hospital stays for patients who have a limited prognosis. Radiation therapy palliates many complications of metastatic cancer and can be completed in a much quicker manner than curative-intent radiation therapy. Unfortunately, palliative radiation in the inpatient setting requires significant time, coordination, and cost, particularly if the radiation facility is located off-site as compared to the hospital. A dedicated CT simulation requires an additional trip to the facility and, usually, additional time, coordination, and resources for transportation and planning. Bypassing CT simulation by planning on a recent diagnostic CT scan, particularly when the scan was obtained within four days prior to consultation, or when treating the spine, can save time, cost, and reduce patient discomfort while expediting palliation.
